# An Anisotropic Peridynamic Model for Simulating Crack Propagation in Isotropic and Anisotropic Rocks

**DOI:** 10.3390/ma16247604

**Published:** 2023-12-12

**Authors:** Kaiwei Tian, Zeqi Zhu, Qian Sheng, Ning Tian

**Affiliations:** 1 State Key Laboratory of Geomechanics and Geotechnical Engineering, Institute of Rock and Soil Mechanics, Chinese Academy of Sciences, Wuhan 430071, China; tiankaiwei96@gmail.com (K.T.); shengqian23@whrsm.ac.cn (Q.S.); tianning19@mails.ucas.ac.cn (N.T.); 2 University of Chinese Academy of Sciences, Beijing 100049, China

**Keywords:** peridynamic, fracture propagation, anisotropy, fracture toughness

## Abstract

In this work, we present a peridynamic-based simulation method for modeling quasi-static fracture propagation in isotropic and anisotropic rock within the framework of peridynamic least square minimization (PDLSM). The original isotropic elastic PDLSM is further extended to investigate fracture propagation in anisotropic materials in this study. The proposed AN-PDLSM model integrates an anisotropic model in fracture mechanics to analyze the failure process of anisotropic rocks. An important advancement in this research lies in the incorporation of the maximum energy release rate criterion (MERR) into the PDLSM framework for the first time. This enhancement enables accurately determining crack propagation and the associated crack angles. The proposed model utilizes the energy release rate calculated through the *J*-integral method to assess bond breakage, and it employs a mesh-independent, piecewise linear fracture model to describe crack propagation. The proposed method fully combines the merits of traditional fracture mechanics with the unique capabilities of peridynamics. To demonstrate the effectiveness of the proposed model, a simulation of fracture evolution in isotropic plates subjected to semi-circular bending tests is presented and compared with experimental results. It is shown that the proposed model accurately replicates fracture trajectories in isotropic specimens. In the context of anisotropic rock, the effect of a weak coefficient on crack morphology is discussed in order to obtain a suitable value. Additionally, the impact of bedding angles on fracture paths through our proposed model is also explored, revealing excellent agreement with experimental results. The findings in this research demonstrate that the proposed AN-PDLSM model is exceptionally proficient at capturing the intricate, oscillating crack paths observed in anisotropic rock materials.

## 1. Introduction

Nowadays, much attention has been paid to isotropic rocks, the strength properties of which are not direction dependent. However, sedimentary rocks, such as shale and coal, are very common in nature and used in different types of rock engineering. Sedimentary rocks always compose interior anisotropic structures formed by geological deposition, thus they should be regarded as anisotropic materials rather than isotropic materials. Crack propagation is the inner mechanism in the failure process of brittle rocks and determines the strength and deformation of rocks, thus it has a great influence on the stability of underground excavation, the efficiency of hydraulic fracture and other geotechnical engineering applications [1]. Consequently, in rock engineering, a thorough understanding of the internal mechanism of fracture propagation in anisotropic rocks is essential. However, this problem is more complicated than isotropic rock because the corresponding formulation must reflect the direction dependence of strength properties in anisotropic rock.

In order to analyze fracture processes in anisotropic materials, numerous numerical methods have been extended to simulate fracture propagation in anisotropic materials. The finite element method (FEM) is used to simulate crack propagation in anisotropic rocks [2]. A 2D anisotropic material model based on the Boundary Element Method (BEM) is proposed to simulate crack propagation in rocks [3]. The extended FEM (XFEM) is further developed to simulate crack propagation in 2D anisotropic media [4]. The lattice models are used to simulate the progressive development of rock failure, starting from micro-crack initiation to their coalescence into larger macro-cracks [5,6]. The phase-field method (PFM) is extended to simulate anisotropic material [7]. The combined finite and discrete element method (FDEM) is extended to model the fracture process in anisotropic rock [8]. Nevertheless, these approaches mentioned above are based on classical (local) continuum mechanics and require additional methods to deal with the spatial discontinuities, i.e., cracks.

Peridynamic (PD) theory proposed by Silling [9] is a kind of non-local mechanics that is found to be more suitable for modeling fracture processes than classical continuum mechanics (CCM) because its governing equations are integral equations rather than partial different equations. There are three types of PD theory, bond-based theory [10], ordinary state-based theory [10] and non-ordinary state-based theory [11]. Bond-based PD has a fixed Poisson’s ratio, whereas the other two types have no restriction. Non-ordinary state-based PD has the obvious advantage of inheriting PD’s ability to deal with cracks while enabling convenient implementations of existing failure models in CCM. The traditional non-ordinary state-based PD, however, suffers from numerical oscillation, which may have a significant impact on simulation results. To overcome this limitation, PD least squares minimization (PDLSM) [12] and the revised non-ordinary state-based PD model have been proposed [13]. PDLSM is an improved non-ordinary state-based PD based on the peridynamic differential operator proposed by Madenci (2016) [14] and has the advantage of efficiently providing commonly used information in CCM such as stress and strain. In the field of geotechnical engineering, peridynamic methods have been used to study the fracture processes in rocks, and good agreement with the experimental results has been reached [15,16].

Anisotropic peridynamic models are firstly proposed by Xu et al. [17] for anisotropic media, but there are some limitations in modeling. Ghajari et al. [18] proposed a bond-based model to remove these limitations, which can be used with any discretization of the domain but still has limitation on the ratio of two critical strain energy release rates. Zhang and Qiao [19] used ordinary state-based PD theory to extend this model and overcome the short-comings, but this model suffers from parameter value uncertainty. Hattori [20] simulated crack propagation in anisotropic solids using the anisotropic failure criterion in the framework of non-ordinary state-based PD. There are grid sensitivity problems because the model adopts the stress-based failure criterion. Tian and Zhou [21] extended the inspired PD model to analyze the fracture development in anisotropic rocks. Nevertheless, this method is affected by surface effects, and cannot effectively display stress, stress intensity factors (SIFs) and other information.

The anisotropy of rock strength is the main factor affecting crack propagation in anisotropic rock [22,23] and it is an effective analytical method to treat anisotropic rocks as rock with anisotropic fracture toughness [24]. Therefore, this paper also conducts research based on this model, and integrates an anisotropic model of the maximum energy release rate criterion (MERR) [25] into the PDLSM framework to analyze the fracture of anisotropic rocks. To the best knowledge of the authors, this is the first time that the anisotropic model MERR has been combined with peridynamics. Considering that the stress-based failure criterion may be strongly influenced by the mesh condition [26] in PD, a mesh-independent bond breakage method based on energy release rate *G*, which is obtained by the *J*-integral [27], is adopted in this paper to avoid this problem. The proposed AN-PDLSM model suited for the anisotropic rock can be easily reduced to an isotropic model and provides an efficient way to analyze crack propagation in both isotropic and anisotropic rocks. Crack propagation in isotropic and anisotropic plates under semi-circular bending tests is simulated based on the proposed model and shows good agreement with the experimental results.

The remainder of this paper is organized as follows. First, the basic theory of PDLSM is introduced in Section 2. The proposed model AN-PDLSM for anisotropic rocks and a description of the numerical implementation is presented in Section 3. Two numerical examples that validate the proposed model and its ability to simulate fracture propagation in both isotropic and isotropic rocks are included in Section 4. Finally, concluding remarks are summarized in Section 5.

## 2. A Brief Introduction to Peridynamic Least Square Minimization (PDLSM)

Peridynamic least square minimization (PDLSM) was first developed by Madenci et al. [12], and combines the concepts of peridynamic differential operator [14] and least square minimization (LSM). In contrast to standard PD theory, PDLSM encompasses the concepts of stress and strain, enabling fully utilizing the constitutive models in traditional continuum mechanics (CCM), as well as the stress intensity factors (SIFs) and the *J*-integral in LEFM to analyze the fracture process. PDLSM is applicable to any arbitrary interaction domain, whereas standard PD theory is limited to sphere interaction domains. As a result, the PDLSM is free of surface effects and volume correction, and it could provide more accurate stress and strain tensor for a wide range of issues compared with standard PD theory.

**Figure 1:**
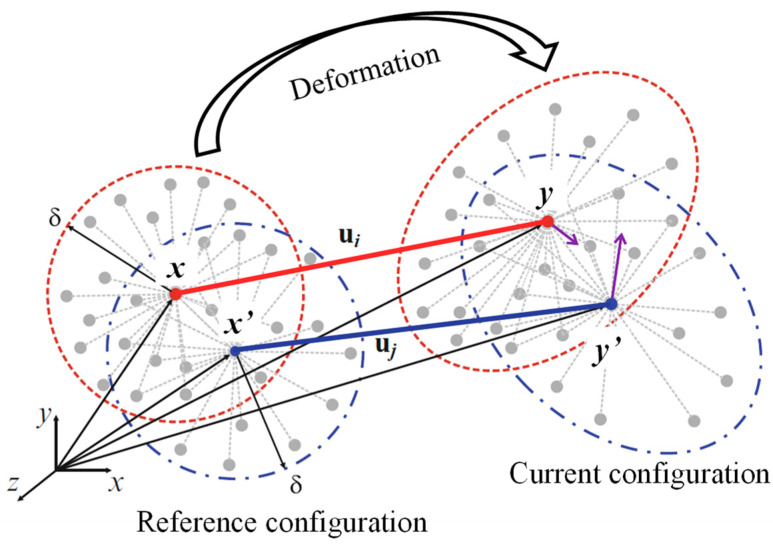
Schematics of peridynamic.

In PDLSM, the equation of motion for linear isotropic material is given by

(1) $$\begin{array}{cc} \rho \left(x\right)\ddot{u}\left(x,t\right) & =L^{pd}+b\left(x,t\right)\\ & =\int_{H_{x}}^{}\omega \left(\left|\xi \right|\right)G\cdot \eta dV_{x^{\prime }}+b(x,t) \end{array}$$

where *ρ* is the mass density, $\ddot{u}$ is the acceleration of node ***x***, ***b*** is the external force vector, and ***ξ*** = ***x***′ − ***x*** and ***η*** = ***u***(***x***′) − ***u***(***x***) are the relative position and relative displacement of nodes ***x***′ and ***x***, respectively, *ω*(|***ξ***|) is the weight function, and the integral part is the internal force vector $L^{pd}$, which is calculated by the integration of bond force between node ***x*** and ***x***′ over the interaction domain *H_x_* of node ***x***, as shown in Figure 1. The matrix ***G*** connects linear elastic Hooke’s law and peridynamic internal force vector $L^{pd}$, according to Liu et al. [26] the matrix ***G*** in the 2D case can be defined for plane stress problems as

(2) $$\left[G\right]=\left[\begin{array}{cc} \frac{E}{2\left(1-\nu \right)}d_{1}+S\left(d_{1}+d_{2}\right) & \frac{E}{2\left(1-\nu \right)}d_{3}\\ \frac{E}{2\left(1-\nu \right)}d_{3} & \frac{E}{2\left(1-\nu \right)}d_{2}+S\left(d_{1}+d_{2}\right) \end{array}\right]$$

and for plane strain problems

(3) $$\left[G\right]=\left[\begin{array}{cc} \left(\lambda +S\right)d_{1}+S\left(d_{1}+d_{2}\right) & \left(\lambda +S\right)d_{3}\\ \left(\lambda +S\right)d_{3} & \left(\lambda +S\right)d_{2}+S\left(d_{1}+d_{2}\right) \end{array}\right]$$

where *λ* is the Lame’s material constant, *S* is the shear modulus, *ν* is Poisson’s ratio, *E* is Young’s modulus, and *d*_1_, *d*_2_, and *d*_3_ are peridynamic differential operators, defined as

(4) $$[\frac{\partial }{\partial x}\;\frac{\partial }{\partial y}\;\frac{\partial^{2}}{\partial x^{2}}\;\frac{\partial^{2}}{\partial y^{2}}\;\frac{\partial^{2}}{\partial xy}]u(x,t)=\int_{H_{x}}^{}\omega \left(\left|\xi \right|\right)[d_{1}\;d_{2}\;{\;d}_{3}\;g_{1}\;g_{2}]\cdot \eta dV_{x^{\prime }}$$

The non-local displacement gradient can be expressed according to the peridynamic differential operator theory,

(5) $$𝛁⨂u(x,t)=\int_{H_{x}}^{}\omega \left(\left|\xi \right|\right)\eta ⨂gdV_{x\prime }$$

where ⨂ is the inner product and ***g*** = [*g*_1_ *g*_2_] is the peridynamic differential operator vector, as explained by Liu et al. [27] and Madenci et al. [12].

According to the CCM, the stain tensor ***ε*** in the framework of infinitesimal deformation is expressed as

(6) $$\varepsilon =\frac{1}{2}(𝛁⨂u+u⨂𝛁)$$

Thus, the stress tensor ***σ*** could also be determined directly based on the strain tensor ***ε*** and the stiffness tensor ***D***, given as

(7) σ=D∶ε

## 3. AN-PDLSM Model for Analyzing Fracture in Anisotropic Rock

In this section, we propose AN-PDLSM, a new method for simulating quasi-static fracture propagation in anisotropic materials, particularly brittle rock, based on the framework of PDLSM and linear elastic fracture mechanics (LEFM). The displacement, strain tensor, and stress tensor are calculated by PDLSM. In this paper, the maximum energy release rate (MERR) criterion proposed by Gao et al. [23] and Wei et al. [22] is adopted to analyze the fracture behavior for anisotropic rocks. In the framework of PDLSM, the MERR criterion is evaluated from stress intensity factors (SIFs), which are calculated from a path-independent *J*-integral. Thus, the proposed method AN-PDLSM makes full use of the existing theories in LEFM to analyze the fracture behavior of anisotropic rocks within the framework of PDLSM, thereby broadening PDLSM’s scope of application.

### 3.1. The Crack Propagation Criterion for Anisotropic Rock

An effective method for studying the fracture behavior of anisotropic rocks is to consider anisotropic rock as rock with anisotropic fracture toughness, which has been widely used in the field of rock mechanics [22,23]. This paper adopts this useful method and combines it with peridynamics in the framework of PDLSM for the first time. In this method, the fracture toughness *G_c_*(*θ*) is assumed to vary with the orientation angle *θ* in anisotropic materials, while remaining constant in isotropic materials. According to the study of Hakim et al. [28], the MERR criterion is extended to materials with anisotropic fracture energy as

(8) $$\underset{\theta }{\max}\frac{G(\theta )}{G_{c}(\theta )}>1$$

where *G*(*θ*) is the energy release rate in different directions.

According to Amestoy and Leblond (1992), the energy release rate *G*(*β*) in a specific direction *β* is defined as

(9) $$G(\beta )=\alpha \left[K_{I}^{2}\left(\beta \right)+K_{II}^{2}\left(\beta \right)\right]$$

Here, *K_I_*(*β*) and *K_II_*(*β*) are kinked stress intensity factors (SIFs) in mode *I* and mode *II* with a deflection angle *β* of the old crack propagation direction, respectively. Parameter *α* is denoted by

(10) $$\alpha =\left\{\begin{array}{c} \frac{\left(1-\upsilon^{2}\right)}{E}\;\;(plane\;strain)\\ \;\;\;\frac{1}{E}\;\;\;\;\;\;\;(plane\;stress) \end{array}\right.$$

The relationship between the kinked SIFs {*K_I_*(*β*), *K_II_*(*β*)} and the SIFs before deflection {*K_I_*, *K_II_*} has been studied by Niusmer [29], and Amestoy and Leblond [30]. In this study, we decide to use the simplest but useful model proposed by Niusmer [29], which is given as

(11) $$K_{I}(\beta )=\frac{1}{2}cos\frac{\beta }{2}[K_{I}\left(1+cos\beta \right)-3K_{II}sin\beta ]\;K_{II}(\beta )=\frac{1}{2}cos\frac{\beta }{2}[K_{I}sin\beta +K_{II}\left(3cos\beta -1\right)]$$

**Figure 2:**
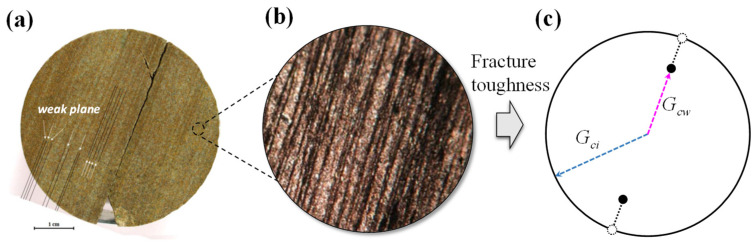
Anisotropic rock with weak plane and corresponding anisotropic fracture toughness *G_c_*. (**a**) anisotropic rock, (**b**) enlarged weak planes, (**c**) anisotropic fracture toughness.

Because the presence of weak planes in anisotropic rocks causes anisotropy in fracture toughness, Gao et al. [23], Zeng and Wei [22] assumed that the rocks have an intact fracture toughness *G_ci_* in all directions except the direction of weak plane, and a weak planar fracture toughness *G_cw_* in the direction of the weak plane, as shown in Figure 2. The weak plane model is utilized in this paper to describe the anisotropic rock, which is given by

(12) $$G_{c}(\theta )=\left\{\begin{array}{c} G_{cw}\;\;(\theta =\gamma \;\;or\;\;\gamma +\pi )\\ \;\;G_{ci}\;\;(\mathrm{other}\;\mathrm{directions}) \end{array}\right.$$

where *G_c_*(*θ*) is the fracture energy in the direction of *θ* and *γ* is the direction of weak plane. It should be noted that this weak plane model can be reduced to the maximum energy release rate criterion for isotropic material by setting *G_cw_* equals to *G_ci_*.

Determining the crack propagation angle *θ_c_* is an important part in analyzing fracture propagation, which is defined as the angle between the direction of old crack and the direction of new crack. The value of *θ_c_* in the isotropic case is denoted by *θ_ci_*, which is calculated by considering the maximum value of the energy release rate *G*(*θ*) in Equation (9), and is found to be

(13) $$\theta_{c}=\theta_{ci}=\left\{\begin{array}{c} 2{tan}^{-1}\left(\frac{K_{I}}{4K_{II}}-\frac{1}{4}\sqrt{{\left(\frac{K_{I}}{K_{II}}\right)}^{2}+8}\;\right)\;\;(if\;K_{II}>0)\\ \;\;2{tan}^{-1}\left(\frac{K_{I}}{4K_{II}}+\frac{1}{4}\sqrt{{\left(\frac{K_{I}}{K_{II}}\right)}^{2}+8}\;\right)\;\;(if\;K_{II}<0) \end{array}\right.$$

However, the value of *θ_c_* in the anisotropic case is obtained in a more complicated way than in the isotropic case because of the existence of the weak planes. And the propagation angle *θ_c_* is calculated according to the MERR criterion for material with anisotropic fracture energy, which is given by

(14) $$\theta_{c}=\left\{\begin{array}{c} {\;\theta }_{ci}\;\;(\;if\;\;\frac{G\left(\theta_{ci}\right)}{G_{ci}}\geq \frac{G\left(\beta \right)}{G_{cw}}\;)\\ \;\;\beta \;\;\;\;(\;if\;\;\frac{G\left(\theta_{ci}\right)}{G_{ci}}<\frac{G\left(\beta \right)}{G_{cw}}\;) \end{array}\right.$$

Once the crack propagation angle *θ_c_* is determined using Equation (14), the condition for determining the crack propagation could be established. If *G*(*θ_c_*) > *G_c_*(*θ_c_*), crack propagate will occur in the direction of *θ_c_*.

According to the author’s knowledge, there are many other anisotropic criteria for the occurrence of cracks for anisotropy solid, such as the anisotropic maximum circumferential stress criterion [31] and anisotropic maximum strain theory [32]. The MERR model used in this article is an anisotropic criterion based on fracture energy. When a crack reaches the critical fracture energy, the crack will propagate. Therefore, this MERR model can effectively consider the occurrence of cracks. Of course, other types of fracture criteria could be considered in future work.

### 3.2. Fracture Energy and the J-Integral

In order to accomplish the MERR within the framework of PDLSM, stress intensity factors (SIFs) and energy release rate *G* must be precisely calculated. Considering that the *J*-integral model is the most common and robust method to obtain the SIFs and is widely used in XFEM [23], the *J*-integral is used to calculate the SIFs in this paper. And the *J*-integral is defined as

(15) $$J=\int_{\Gamma }^{}\left(\frac{1}{2}\sigma_{ij}\varepsilon_{ij}\delta_{1k}-\sigma_{ik}\frac{\partial u_{i}}{\partial x_{1}}\right)n_{k}d\Gamma$$

in which *δ* is the Kronecker delta, *σ_ij_* is the stress tensor, *ε_ij_* is the strain tensor, *u_k_* is the displacement vector, and *n_k_* is the unit outward normal vector on the integration contour *Γ*.

In the field of linear elastic fracture mechanics (LEFM), the *J*-integral is equal to the energy release rate *G*, and is related to the SIFs *K_I_* and *K_II_* by the well-known relationships,

(16) $$J=G=\alpha {(K}_{I}^{2}+K_{II}^{2})$$

where the SIFs *K_I_* and *K_II_* can be directly determined by solving two equations based on the interaction integral (*I*-integral) theory proposed by Yau et al. [33],

(17) $$\left\{\begin{array}{c} M^{\left(1,2a\right)}={2\alpha K}_{I}\\ \;M^{\left(1,2b\right)}={2\alpha K}_{II} \end{array}\right.$$

where *M*^(1,2*a*)^ and *M*^(1,2*b*)^ are two interaction integrals defined as

(18) $$\left\{\begin{array}{c} M^{\left(1,2a\right)}=\int_{\Gamma }^{}\left(\sigma_{ij}^{\left(1\right)}\varepsilon_{ij}^{\left(2a\right)}\delta_{1j}-\sigma_{ij}^{\left(1\right)}\frac{\partial u_{i}^{\left(2a\right)}}{\partial x_{1}}-\sigma_{ij}^{\left(2a\right)}\frac{\partial u_{i}^{\left(1\right)}}{\partial x_{1}}\right)n_{j}d\Gamma \\ M^{\left(1,2b\right)}=\int_{\Gamma }^{}\left(\sigma_{ij}^{\left(1\right)}\varepsilon_{ij}^{\left(2b\right)}\delta_{1j}-\sigma_{ij}^{\left(1\right)}\frac{\partial u_{i}^{\left(2b\right)}}{\partial x_{1}}-\sigma_{ij}^{\left(2b\right)}\frac{\partial u_{i}^{\left(1\right)}}{\partial x_{1}}\right)n_{j}d\Gamma \end{array}\right.$$

{$\sigma_{ij}^{\left(1\right)}$, $u_{i}^{\left(1\right)}$} denote the actual solution of stress tensor and displacement vector calculated by PDLSM; {$\varepsilon_{ij}^{\left(2a\right)}$, $u_{i}^{\left(2a\right)}$, $\sigma_{ij}^{\left(2a\right)}$} are solutions of strain tensor, displacement vector, stress tensor under the assumption of *K_I_* = 1 and *K_II_* = 0; and {$\varepsilon_{ij}^{\left(2b\right)}$, $u_{i}^{\left(2b\right)}$, $\sigma_{ij}^{\left(2b\right)}$} are solutions of strain tensor, displacement vector, stress tensor under the assumption of *K_I_* = 0 and *K_II_* = 1. The detailed description of those parts could be found in Yau’s [33] paper.

**Figure 3:**
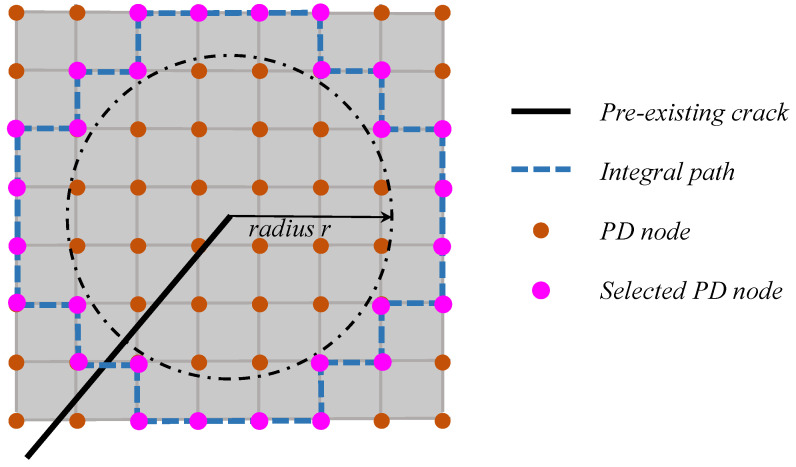
A schematic diagram of the *J*-integral in PDLSM. (Modified form [26]).

From a numerical perspective, an integration contour path should be specified to obtain the *J*-integral. The numerical integration method proposed by Liu et al. [26] is employed in this study, as shown in Figure 3. The selected PD nodes and integral path for calculating the *J*-integral could be de determined if the integral radius *r* is specified, which should be a suitable value, usually 6 fold the minimal node space. With the integral path from the selected PD nodes, the *J*-integral could be calculated using Equation (16).

### 3.3. A Mesh-Independent Piecewise Linear Model for Bond Breakage

The traditional XFEM, CZM and FDEM methods usually limit fracture propagation along the element boundaries and could only deflect at the element boundaries. Obviously, those methods are sensitive to the mesh condition and require fine mesh to reduce this effect, while fine mesh will significantly increase the computational burden.

**Figure 4:**
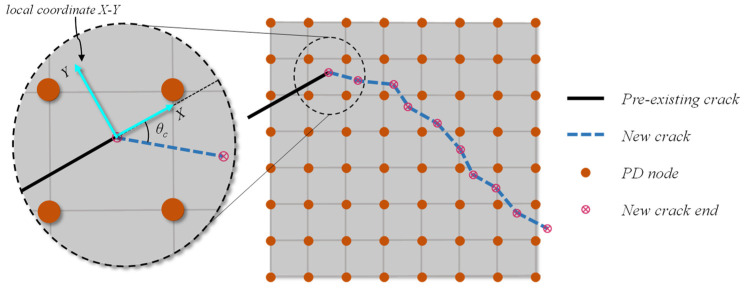
A schematic diagram of mesh-independent piecewise linear fracture growth.

Peridynamic bonds are assumed to be broken when they reach a certain state, and the fracture process is accomplished by breaking the bonds that connects two PD nodes. Existing studies have shown that this bond breakage method also exhibits grid dependencies [34]. In address this shortcoming, a mesh-independent piecewise linear model for bond breakage is adopted in this paper to simulate the fracture process of rocks, as shown in Figure 4. Once the new crack is determined using the MERR criterion described in Section 3.1, the bonds intersected with the new crack are set to break and the interaction force on the bond vanishes. This bond breaking model differs from the original bond breaking manner. As mentioned by Bazant et al. [35], the original bond breakage method may cause an unreal phenomenon since the PD nodes, on opposite sides of the crack, may have ghost interaction in some cases. On the contrary, this mesh-independent piecewise linear model could eliminate all interactions, making it more physical, and is adopted by some researchers like Liu et al. [26] and Ni et al. [36].

In the proposed model, the new crack could propagate in any direction, not limited to the element boundaries. In numerical simulation, the fracture propagation direction can be determined by the LEFM exactly, whereas the fracture growth length is chosen in a somewhat arbitrary way. The fracture growth length is set to be proportional to the sqrt of minimum area of PD nodes according to Liu et al. [26].

(19) $${∆}_{min}=k\sqrt{A_{min}}$$

where *A_min_* is the minimum area of PD nodes and *k* is a fracture growth length parameter, which is set to be 1.0 in this study.

A scalar variable *μ*(***ξ***,*t*) is used in the AN-PDLSM model to record the damage state of the bonds varying with time. Once the bond reaches a critical state, the bond is set to be broken, and the force on the bond vanishes. The scalar variable *μ*(***ξ***,*t*) is defined as

(20) $$\mu (\xi ,t)=\left\{\begin{array}{c} 1\;\;(\mathrm{if}\;\mathrm{the}\;\mathrm{bond}\;\mathrm{is}\;\mathrm{intact})\\ \;\;\;0\;\;(\mathrm{if}\;\mathrm{the}\;\mathrm{bond}\;\mathrm{is}\;\mathrm{broken}) \end{array}\right.$$

A damage variable *D* is fined on the node level rather than the bond level in order to conveniently display the damage of the entire body. The damage *D*(***x***,*t*) of a node ***x*** is calculated by the proportion of broken bonds and intact bonds in the corresponding interaction domain *H_x_*, which is defined as

(21) $$D\left(x,t\right)=1-\frac{\int_{H_{x}}^{}\mu (\xi ,t)dV_{x^{\prime }}}{\int_{H_{x}}^{}dV_{x^{\prime }}}$$

### 3.4. Implicit Solver of AN-PDLSM

The quasi-static fracture problem is widely studied in the fields of rock mechanics and rock engineering, and this paper aims to model a quasi-static fracture process rather than a dynamic fracture process. The explicit local damping method [37] and the adaptive dynamic relaxation method (ADR) [38] are widely used and preferred for quasi-static problems. However, compared to those explicit methods, the implicit algorithm is better suited for quasi-static problems in some cases because it does not require satisfying the C-F-L condition and is unconditionally convergent [36].

Considering the foregoing, this paper employs the implicit method to solve the governing equation, i.e., the equilibrium equations. The weak form of AN-PDLSM is obtained by applying the weighted residual method (WRM) on the equation of motion and the boundary conditions (B.C.), which is given as

(22) $$\int_{V_{pd}}^{}\delta u\cdot (L_{pd}+b-\rho \ddot{u})dV-\int_{S_{pd}}^{}\delta u\cdot (\sigma_{pd}\cdot n-T)dS=0$$

where ***T*** is the external traction on the boundary, ***n*** is the unit normal vector of the boundary, and δ***u*** is a virtual displacement. *V_pd_* means the volume of the entire body, and *S_pd_* is the boundary of the body.

According to the study of Liu et al. (2021b), the matrix form of the governing Equation (22) can be written in the same form as FEM, which is given as

(23) $$M^{pd}\ddot{u}\;+K^{pd}u=F^{pd}$$

and the global stiffness matrix ***K****^pd^* is defined as

(24) $$K^{pd}=K_{b}^{pd}+K_{s}^{pd}$$

where ***u*** is the nodal displacement vector, ***F****^PD^* is the global equivalent node force, and **M***^PD^* is the global equivalent nodal mass. In the AN-PDLSM model, the global stiffness matrix ***K****^PD^* consists of two parts, ***K_b_****^PD^* associated with PD domain and ***K_s_****^PD^* associated with PD boundary. The stiffness matrix ***K****^PD^* varies with the bond breakage state. The matrix will be updated if any bond is set to be broken, and the corresponding stiffness of the broken bond vanishes. The detailed description of the global stiffness matrix ***K****^PD^* could refer to the study of Liu [26] and Medanci [12].

The global displacement vector ***u*** can be expressed as

(25) $$u=\left[\begin{array}{c} u_{u}\\ u_{p} \end{array}\right]$$

Here, ***u****_u_* is the set of all unknown displacement components and ***u****_p_* is the set of prescribed displacement components. For static or quasi-static problems, the acceleration vector $\ddot{u}$ is negligibly small, the dynamic term $M^{pd}\ddot{u}\;is\;vanished$. Thus, governing Equation (23) can be reduced to

(26) $$\left[\begin{array}{cc} K_{uu} & K_{up}\\ K_{pu} & K_{pp} \end{array}\right]\left[\begin{array}{c} u_{u}\\ u_{p} \end{array}\right]=\left[\begin{array}{c} F_{u}\\ F_{p} \end{array}\right]$$

Since ***u****_p_* is the prescribed displacement constraint, the governing Equation (26) for quasi-static problems reduces to

(27) $$K_{uu}u_{u}=F_{u}-K_{up}u_{p}$$

By solving the above large linear systems of equations, the unknown displacement components ***u****_u_* can be solved.

In this paper, these large linear systems of equations are solved by the Parallel Direct Sparse Solver of Intel Math Kernel Library (MKL). Compared to the explicit dynamic relaxation method, directly and implicitly solving Equation (27) avoids a large number of iterations and is highly computationally efficient. The direct solving method based on LU decomposition is selected to solve the large linear systems of equations. The large sparse matrix is stored in a compressed sparse row (CSR) format, which is one of the inputs of the Parallel Direct Sparse Solver. The above numerical calculation process is implemented using code written in C++.

Generally, the explicit dynamic relaxation method requires a large number of iterations for each time step to reach a convergent state. Compared to the explicit ADR method, the implicit method does not need a large number of iterations for solutions, and convergent is guaranteed in each times step. Although the implicit method requires to solve the inversion of matrix ***K***^PD^, the stiffness matrix ***K***^PD^ is symmetric and sparse and its inversion is relatively easier and fast computationally using the Parallel Direct Sparse Solver.

In the traditional PD theory, applying boundary conditions is much more complex than in FEM. In those PD theories, a fictitious layer with non-zero volume is required for applying boundary conditions. However, within the framework of PDLSM, the constraint and traction boundary conditions can be treated the same way as the FEM method, making it more convenient than traditional PD theories. And those feathers are the advantages of the AN-PDLSM method compared with classic PD theory.

The implicit numerical implementation is divided into seven steps, step 1 is to build the computing mesh model; Step 2 is to input calculation parameters; Step 3 is to apply boundary conditions; Step 4 is to integrate the stiffness matrix ***K****^pd^* and node force vector ***F****^pd^*; Step 5 is to solve linear algebraic equations for displacement, strain and stress; Step 6 is to calculate SIFs and the energy release rate *G* according to the *J*-integral; Step 7 is to calculate the crack propagation angle *θ_c_* and the corresponding energy release rate *G*(*θ_c_*); Step 8 is to determine the bond breakage condition and break the bond according to the MERR.

In Step 1 of establishing a computational mesh, the traditional PD often uses uniform mesh, but will greatly increase the amount of computation because it divides the mesh of unimportant areas as small as the areas of interest. On the contrary, the proposed AN-PDLSM can use non-uniform mesh, with fine mesh in the regions of interest and coarse mesh in the less important regions. Previous studies have shown that PDLSM has grid-independent properties, and the proposed AN-PDLSM also inherits this advantage, which is superior to the traditional PD method [12]. All the computational meshes in this paper are generated by commercial software Abaqus 2020, but only nodal information is used, while information of elements is not required, because AN-PDLSM is still a meshless method in essence.

## 4. Numerical Verification and Simulation

Two plane stress examples are performed to demonstrate the capability of the proposed AN-PDLSM model to simulate fracture propagation in isotropic and anisotropic rocks. The first example verifies the fracture propagation of an isotropic plate under a semi-circular bending test, while the second example simulates the fracture process of a anisotropic semi-circular bending specimen and discusses the influence of a weak coefficient and a weak planar direction on the crack trajectory.

### 4.1. Fracture Analysis of Isotropic Semi-Circular Bending Tests

**Figure 5:**
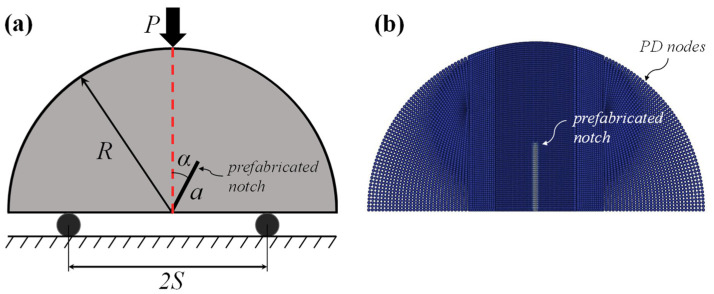
(**a**) Schematic of a semi-circular bending specimen with a prefabricated notch; (**b**) non-uniform PD mesh.

As a benchmark problem, an isotropic semi-circular bending specimen made of polymethylmethacrylate (PMMA) with a prefabricated notch is simulated to validate the capability of the AN-PDLSM model to capture crack evolution in isotropic materials, as shown in Figure 5. If the material is isotropic, the AN-PDLSM model is reduced to the PDLSM model, as described in Section 3. The isotropic semi-circular bending plate is subjected to displacement loading *u*, which increases from zero with incremental Δ*u* =1.6 × 10^−5^ m on the top of the numerical model. The displacement of the left support pin in the *x* and *y* directions is fixed, while the *y* direction of the right pin is fixed and the *x* direction is free. The angle formed by the prefabricated notch and the vertical line is denoted by *α* (Figure 5a). The dimensions of the specimen are determined as radius *R* = 50 mm, crack length *a* = 15 mm, support distance 2*S* = 43 mm, and thickness *t* = 5 mm. The mechanical properties of PMMA are specified as Young’s modulus *E* = 3.75 GPa, Poisson’s ratio *ν* = 0.31, and fracture toughness *K*_IC_ = 2.13 MPa·m^1/2^.

As shown in Figure 5b, the entire domain is discretized with 10,512 PD nodes with a non-uniform mesh rather than uniform mesh, with the mesh of the middle part of the specimen being finer than the edge part. The size of the horizon is specified as *δ* = 3*d*, where *d* is the PD node space. The corresponding numerical simulation process is carried out as described in Section 3.4. Since the sample is isotropic, the intact fracture toughness *G_ci_* is equal to the weak planar fracture toughness *G_cw_*, and the crack propagation angle *θ_c_* is calculated using Equation (13).

**Figure 6:**
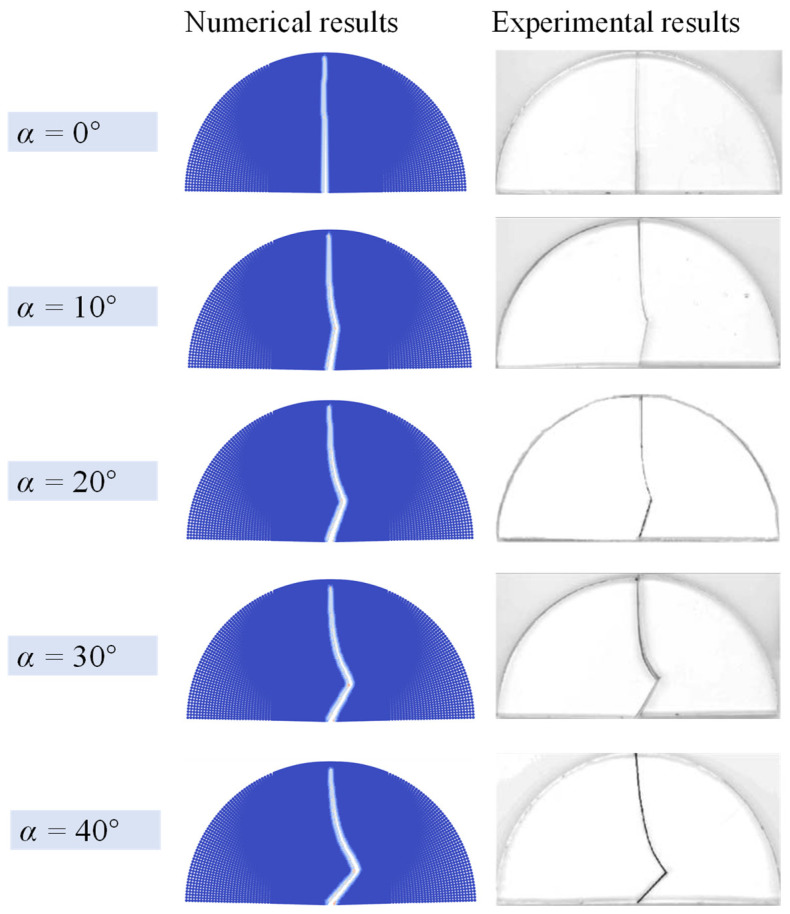
Comparison of numerical results and experimental results [39] of isotropic specimens with different prefabricated notch angle *α* under semi-circular bending tests.

Figure 6 illustrate the numerical results of five specimens with different angles *α* = 0°, 10°, 20°, 30°, 40°. When *α* = 0°, the fracture propagates along the direction of the prefabricated notch, resulting in pure model I fracture pattern. When *α* is set to other angles, the fracture propagate propagates as a wing crack from the notch tip and eventually aligns with the horizontal direction. The fracture trajectories of the five cases clearly agree with the experiment results of Ayatollahi et al. [39]. Consequently, the proposed AN-PDLSM model can be reduce to the isotropic case and effectively simulate the fracture process of an isotropic rock under a semi-circular bending test.

**Figure 7:**
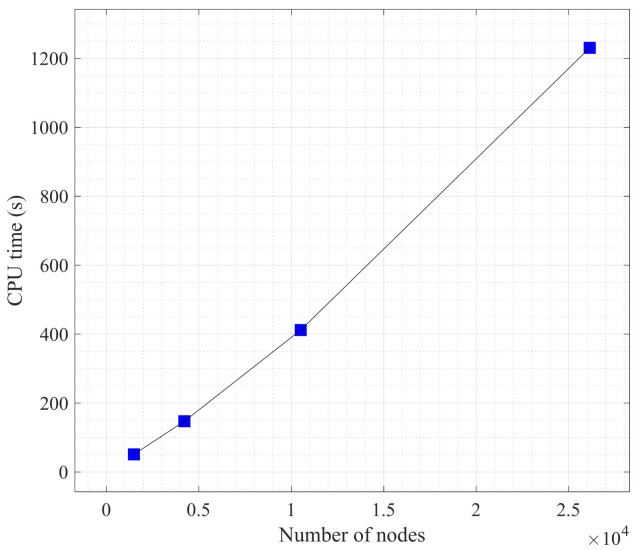
Computation time (CPU time) for simulation with different mesh sizes.

In order to analyze the robustness of the proposed model and examine the influence of mesh size on the calculation results. Semi-circular bending tests with different mesh sizes are simulated, respectively, and the notch angle *α* of the specimens is set to 30°. Because non-uniform meshes are used in this paper, the number of nodes is used to represent the size of the mesh. Four cases with different mesh size are set, as shown in Table 1. To ensure a single variable, the time steps of the four numerical cases are set to 350 steps. Table 1 shows the calculation time (CPU time) for different cases, and Figure 7 plots the relationship between the calculation time and the number of nodes. The results show that the calculation time has an approximately linear relationship with the number of nodes.

**Figure 8:**
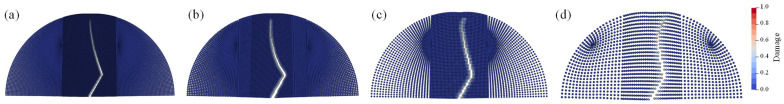
Comparison of numerical results with different mesh size. (**a**) mesh with 26,138 nodes, (**b**) mesh with 10,512 nodes, (**c**) mesh with 4218 nodes, (**d**) mesh with 1490 nodes.

Figure 8 shows the crack shapes corresponding to different mesh size. It can be found that the crack shapes of the four cases are generally consistent. The results suggest that the mesh size does not have a significant impact on the numerical results. Even coarser meshes simulate crack shapes well and achieve good results. According to the author’s experience, to obtain the best simulation results, the grid distance d*x* should be smaller than 1/100 of the length of the sample.

### 4.2. Fracture Analysis of Anisotropic Semi-Circular Bending Tests

#### 4.2.1. Influence of a Bedding Weak Coefficient

In this section, the proposed AN-PDLSM model described in Section 3 is used to analyze the crack propagation of anisotropic rock subjected to a semi-circular bending test. An anisotropic semi-circular plate is subjected to displacement loading *u* increasing from zero with an incremental Δ*u* = 1.6 × 10^−5^ m on the top of the specimen. The dimensions and the boundary conditions of this specimen are determined in the same way as the isotropic model described in Section 4.1. The mechanical properties of the specimen are specified according to the study of Wang and Teng. [40] as *E* = 2326.31 MPa, *ν* = 0.31, and intact fracture toughness *G_ci_* = 20 J. The entire domain is discretized with 10,512 PD nodes, the same as the numerical model in Section 4.1. The size of the horizon is specified as *δ* = 3*d*, where *d* is the PD node space.

In the anisotropic model, the fracture toughness *G_c_* is no longer constant, but contains two different values *G_cw_* and *G_ci_*. For the convenience of analysis, the ratio of *G_cw_* and *G_ci_* is defined as the bedding weak coefficient *η* = *G_cw_/G_ci_*, which reflects the degree of softening of the bedding relative to the intact rock. The bedding weak coefficient *η* = 1 indicates that the bedding is consistent with the intact rock, and *η* close to 0 indicates that the bedding is very weak. In order to quantitatively evaluate the influence of a weak coefficient *η* on the fracture pattern of rock under a semi-circular bending test, several simulations were carried out in the case of *η* = 0.5, 0.6, 0.7, 0.8, 0.9, 1.0, and other variables remained unchanged. The Angle of prefabricated notch was 0°, crack length a is equal to 15 mm and the direction of the bedding is π/6.

**Figure 9:**
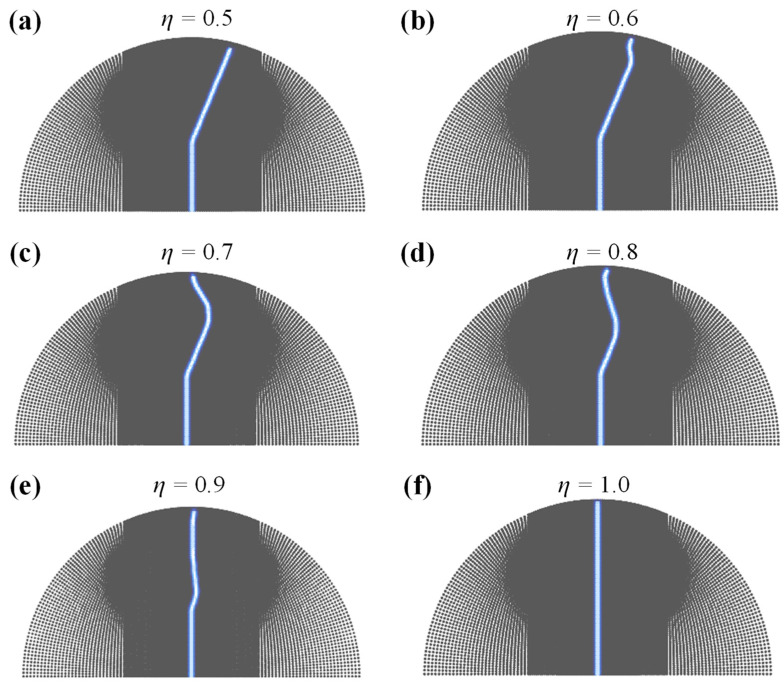
The fracture process of a semi-circular bending specimen with different *G*_0_/*G*_w_ ratios, that is the weak coefficient *η*.

Figure 9 illustrate the fracture trajectory of specimens with a different weak coefficient *η*. The numerical results show that *η* has a significantly effect on the morphology of fracture propagation in a semi-circular bending test. When *η* = 0.5, the bedding strength is much lower than the intact rock, and the fracture extends completely along the bedding direction. When *η* = 1.0, the bedding strength is equal to that of intact rock, i.e., there is no bedding, and the fracture propagates vertically upward completely, which is consistent with the isotropic case in Section 4.1. When *η* is between 0.5 and 1.0, the fracture presents a typical two-stage expansion, with the crack first expanding along the bedding, which is referred to as bedding crack in this paper, and then deviating from the bedding direction to the vertical line, which is referred to as wing crack in this paper. Bedding crack represents crack along the bedding, while wing crack represents fracture that occurs in intact rock.

**Figure 10:**
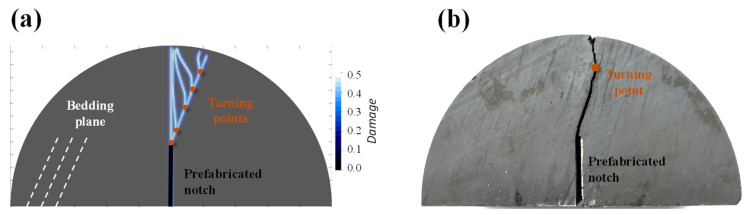
(**a**) The crack trajectory of a plate under a semi-circular bending test with prefabricated notch angle *α* = 90°, bedding angle *θ* = 67.5°, The weak coefficients *η* are, respectively, 1.0, 0.9, 0.8, 0.7, 0.6, 0.5. (**b**) Experimental results [40].

Figure 10 depicts a comparison of the crack morphology and experiment results for various weak coefficients *η*. The turning point is the point at which the crack transitions from bedding crack to wing crack. As *η* decreases from 1.0 to 0.5, the turning point appears closer to the top part of the specimen; The proportion of bedding cracks increases while the proportion of wing crack decreases. Compared with the experiment results by Wang and Teng. [40], it is clear that when *η* equals to 0.6, the numerical fracture path is basically consistent with the experimental results. Thus, *η* = 0.6 is selected to describe anisotropic rock in the proposed AN-PDLSM model in the following part.

**Figure 11:**
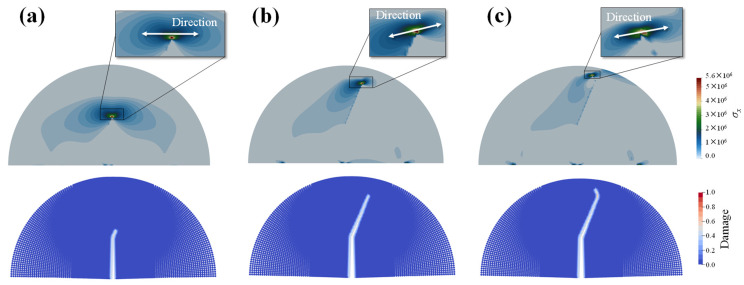
The distribution of stress component *σ_x_* of different failure stage in the case of *η* = 0.6. (**a**) crack initiation, (**b**) reach turning point, (**c**) wing crack development.

Unlike the bond-based peridynamic model, which lacks the definition of stress tensor, AN-PDLSM contains the concept of stress, enabling it to analyze the evolution of stress during the failure process. Because horizontal tensile failure will occur in the rock sample a under semi-circular bending test, we provide the contour of horizontal stress *σ_x_* corresponding to the failure process of plate under loading when *η* = 0.6, as shown in Figure 11. The tensile stress *σ_x_* at the crack tip presents a symmetrical distribution when the fracture begins to propagate, as shown in Figure 11a; When the fracture reaches the turning point, the stress distribution at the crack tip changes, the direction of the stress deflates, and the crack type changes from bedding crack to wing crack. The stress symmetry axis of the wing crack tip is generally perpendicular to the crack.

**Figure 12:**
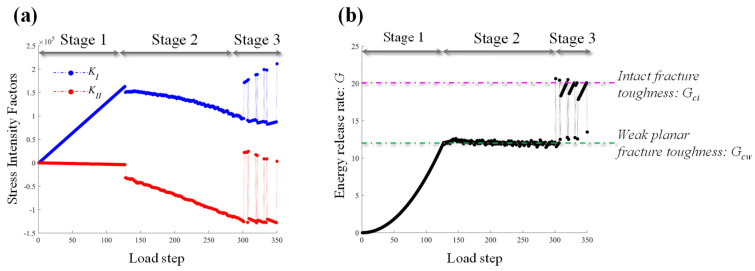
Three typical loading stages and corresponding (**a**) stress intensity factor, SIFs; (**b**) energy release rate, *G*.

In order to analyze the mechanism of the turning point, the SIFs {*K_I_*, *K_II_*} and energy release rate *G* of each time step have been recorded, as shown in Figure 12. The loading stage can be divided into three different stages based on the variation in energy release rate *G* during the loading process. The first stage is the loading stage without crack propagation. The numerical results show that at this stage, the *K*_I_ increases linearly under loading, whereas the *K_II_* remains zero. Thus, the fracture pattern in stage one is type *I* crack mode in this test. The energy release rate *G* increases in the form of a quadratic function, which is consistent with Formula (16). The second stage is the bedding crack propagation stage. Figure 12b shows that the energy release rate *G* in this stage is fixed around *G_cw_*, which is equal to η*G_ci_, indicating the accuracy of the calculated results. In the second stage, the SIFs *K_I_* and *K_II_* decreases almost linearly, respectively. The third stage corresponds to the wing crack propagation stage. In this stage, most of the energy release rate *G* equals to *G_ci_*, while only a small part equals to *G_cw_*. It implies that the predominant crack at this stage is the wing crack, with the bedding crack making up a much smaller portion.

When compared to the isotropic case in Section 4.1, the crack trajectory of anisotropic model is clearly more complicated than that of the isotropic model due to the presence of the oscillation cracks and transition zones, which have been reported in anisotropic rocks [41]. According to the experimental results of Wang and Teng [40], the fracture starts from the notch tip and develops along the bedding angle before deflecting in the opposite direction in the transition zone, producing the intricate oscillation cracks depicted in Figure 10b. The proposed AN-PDLSM model captures this complex crack path well, demonstrating the method’s effectiveness in simulating crack propagation in anisotropic rock under a semi-circular bending test.

#### 4.2.2. Influence of Bedding Plane Angle

On the basis of Section 4.2.1, the proposed AN-PDLSM model is used to analyze the influence of the bedding angle on the fracture trajectory. The specimen with 5 different bedding angles 0°, 22.5°, 45°, 67.5°, and 90° are simulated, respectively. Except for the bedding angle, the other parameters are consistent with Section 4.2.1. It should be noted that in those simulations, the weak coefficient *η* is set to 0.6.

**Figure 13:**
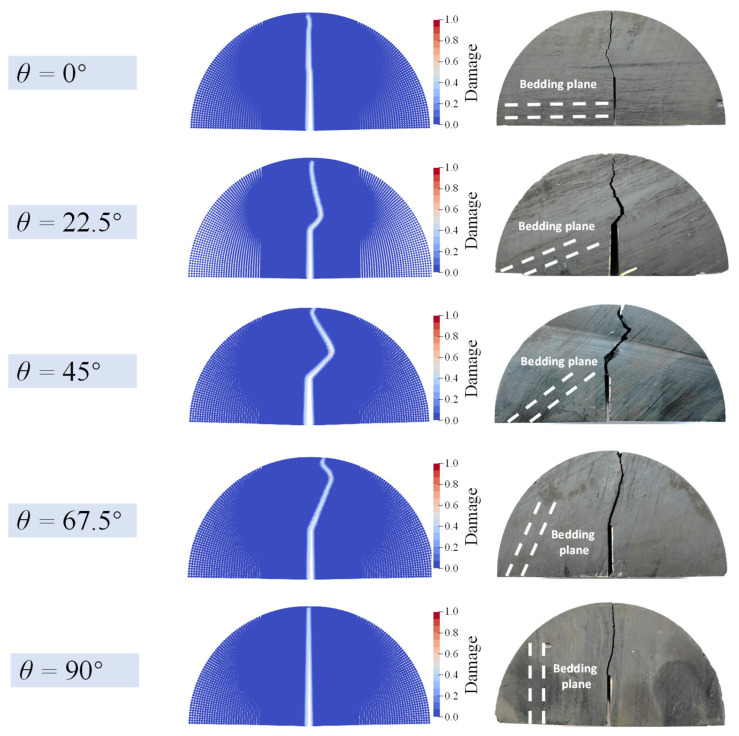
Comparison of numerical crack trajectory and experimental crack path (Wang et al., 2022) of an anisotropic semi-circular bending specimen with prefabricated notch angle *α* = 0° and different bedding angle *θ*.

All of those numerical results are compared with the experimental results, respectively, as shown in Figure 13. The numerical results are found to be in good agreement with the experimental results. The crack propagates vertically upward when the bedding angle *θ* is equal to 0° and 90°, respectively. When the bedding angle *θ* is equal to 22.5°, 45° and 67.5°, the crack path appears as a complex oscillating crack; In the experimental results, the oscillating crack first propagates along the bedding before converting to a wing crack after reaching the turning point. Furthermore, as the bedding angle *θ* increases from 22.5° to 67.5°, the length of the bedding crack increases; while the length of wing crack decreases.

**Figure 14:**
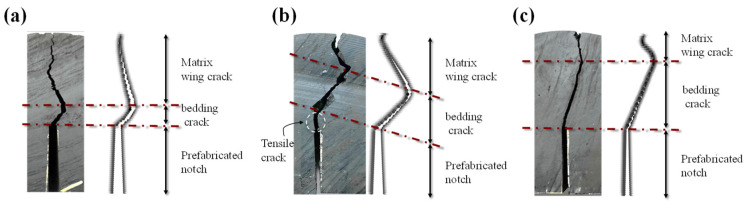
Comparison of numerical crack length and experimental results [40] of an anisotropic semi-circular bending specimen with prefabricated notch angle *α* = 0° (**a**) bedding angle *θ* = 22.5° (**b**) bedding angle *θ* = 45° (**c**) bedding angle *θ* = 67.5°.

Figure 14 demonstrates a detailed comparison of the crack length between the numerical results and the experimental results. The length of the simulated bedding crack is consistent with the experiment, and the crack propagation angles of bedding crack and wing crack are in good agreement, respectively. There is a slight discrepancy between numerical results and experimental results in the case of bedding angel equal to 45°, since there occurs a vertical tension crack at the crack tip before entering the bedding crack. Regardless, the numerical simulation precisely captured the bedding crack and wing crack following the tensile crack. The proposed AN-PDLSM model can accurately simulate this complex oscillating crack and the length of the bedding crack that varies with the bedding angle.

## 5. Conclusions

In this study, peridynamic least square minimization (PDLSM) has been extended to anisotropic materials named AN-PDLSM model, based on the weighted residual method. The proposed AN-PDLSM model integrates an anisotropic model of fracture mechanics to analyze the failure process of anisotropic rocks. The rock matrix and bedding part are assumed to possess different levels of fracture toughness, and the improved maximum energy release rate criterion (MERR) is used to determine crack propagation condition and the angle of crack. In addition, the proposed AN-PDLSM model can be easily reduced to the isotropic PDLSM model. The proposed AN-PDLSM could apply boundary conditions the same as FEM and does not require surface correction and volume correction, thus improving computation accuracy. To simulate crack propagation, bonds are interrupted by a grid-independent piecewise linear model. In terms of numerical accomplishments, the proposed model uses an implicit method for quasi-static problems.

Crack propagation in isotropic plates during semi-circular bending testing is simulated based on the suggested model in order to confirm its accuracy. The numerical results show good agreement with the experimental results, confirming the effectiveness of the proposed model for analyzing fracture in isotropic rock and its ability to be reduced to the isotropic PDLSM model.

For anisotropic rocks, crack propagation in anisotropic plates under semi-circular bending tests is simulated, and the influence of the bedding weak coefficient *η* on the crack morphology is analyzed. The value of *η* has an impact on the location of a turning point. The proportion of bedding cracks increases with the decrease in the value of *η*, while the proportion of wing crack decreases, and vice versa. The highest agreement between the simulated and experimental results occurs when *η* = 0.6, which is a suitable value to characterize the weak degree of the bedding of the anisotropic rock analyzed in this paper. By analyzing the stress distribution of the failure process, it is found to be symmetrical before reaching the turning point, the stress symmetry axis will turn after the turning point. The mechanism of bedding crack and wing crack are examined by analyzing the stress intensity factor SIFs and energy release rate *G*, respectively. The rationality of the proposed numerical model is further verified by those analyses.

Additionally, a simulation of rock failure with various bedding angles is performed and the results are in good agreement with the experimental findings. Simple vertical cracks form at bedding angles of 0° and 90°; complex oscillating cracks appear at bedding angles of 22.5°, 45°, and 67.5°. For oscillating cracks, the crack morphology, bedding crack length, and wing crack length simulated by the model are quite similar to the experimental results. The results demonstrate the proposed AN-PDLSM model’s capacity to capture the complex crack path. In conclusion, the proposed AN-PDLSM model can accurately simulate fracture propagation in isotropic and isotropic rocks.

## Figures and Tables

**Table 1:** The CPU time for simulation with different mesh sizes.

	Case 1	Case 2	Case 3	Case 4
Number of nodes	26,138	10,512	4218	1490
CPU time (s)	1230.91	411.93	147.14	50.95

## Data Availability

Data are contained within the article.
